# Imported Case of Acute Respiratory Tract Infection Associated with a Member of Species *Nelson Bay Orthoreovirus*

**DOI:** 10.1371/journal.pone.0092777

**Published:** 2014-03-25

**Authors:** Atsushi Yamanaka, Akira Iwakiri, Tomoki Yoshikawa, Kouji Sakai, Harpal Singh, Daisuke Himeji, Ikuo Kikuchi, Akira Ueda, Seigo Yamamoto, Miho Miura, Yoko Shioyama, Kimiko Kawano, Tokiko Nagaishi, Minako Saito, Masumi Minomo, Naoyasu Iwamoto, Yoshio Hidaka, Hirotoshi Sohma, Takeshi Kobayashi, Yuta Kanai, Takehiro Kawagishi, Noriyo Nagata, Shuetsu Fukushi, Tetsuya Mizutani, Hideki Tani, Satoshi Taniguchi, Aiko Fukuma, Masayuki Shimojima, Ichiro Kurane, Tsutomu Kageyama, Takato Odagiri, Masayuki Saijo, Shigeru Morikawa

**Affiliations:** 1 Department of Internal Medicine, Miyazaki Prefectural Miyazaki Hospital, Miyazaki, Miyazaki, Japan; 2 Department of Microbiology, Miyazaki Prefectural Institute for Public Health and Environment, Miyazaki, Miyazaki, Japan; 3 Special Pathogens Laboratory, Department of Virology 1, National Institute of Infectious Diseases, Musashimurayama, Tokyo, Japan; 4 Nichinan Public Health Office of Miyazaki Prefecture, Nichinan, Miyzakaki, Japan; 5 Miyazaki City Public Health Office, Miyazaki, Miyazaki, Japan; 6 Health Promotion Division, Miyazaki Prefecture Government, Miyazaki, Miyazaki, Japan; 7 Laboratory of Viral Replication, International Research Center for Infectious Diseases, Research Institute for Microbial Diseases, Osaka University, Suita, Osaka, Japan; 8 Department of Pathology, National Institute of Infectious Diseases, Musashimurayama, Tokyo, Japan; 9 Influenza virus Research Center, National Institute of Infectious Diseases, Musashimurayama, Tokyo, Japan; 10 Department of Veterinary Science, National Institute of Infectious Diseases, Shinjuku, Tokyo, Japan; Faculty of Biochemistry Biophysics and Biotechnology, Jagiellonian University, Poland

## Abstract

A Japanese man suffered from acute respiratory tract infection after returning to Japan from Bali, Indonesia in 2007. Miyazaki-Bali/2007, a strain of the species of *Nelson Bay orthoreovirus*, was isolated from the patient's throat swab using Vero cells, in which syncytium formation was observed. This is the sixth report describing a patient with respiratory tract infection caused by an orthoreovirus classified to the species of *Nelson Bay orthoreovirus*. Given the possibility that all of the patients were infected in Malaysia and Indonesia, prospective surveillance on orthoreovirus infections should be carried out in Southeast Asia. Furthermore, contact surveillance study suggests that the risk of human-to-human infection of the species of *Nelson Bay orthoreovirus* would seem to be low.

## Introduction

Genus *Orthoreovirus* is one of 15 current genera in Family *Reoviridae* comprises nonenveloped virus with segmented double stranded (ds) RNA genomes that are taxonomically classified into 15 genera, each containing 10 genome segments. Orthoreoviruses have been isolated from a variety of mammalian, avian, reptilian, and piscine hosts. Orthoreoviruses are divided into the fusogenic and nonfusogenic subgroups, based on the ability of the virus to induce cell-to-cell fusion and syncytium formation [1]. The majority of orthoreoviruses are fusogenic, including avian, baboon, reptilian and *Nelson Bay* orthoreoviruses; while the mammalian and piscine orthoreoviruses (MRV) are nonfusogenic. In humans, MRV infections are common, but are usually asymptomatic or mildly symptomatic [1], [2]. In the last decade, however, several orthoreoviruses, which seem to have originated in fruit bats including the grey-headed flying fox (*Pteropus poliocephalus*) and small flying fox (*Pteropus hypomelanus*), have been identified as causative agents for respiratory tract infection (RTI) in Malaysia and Indonesia [3]–[6]. Another orthoreovirus, Xi River virus, was isolated from fruit bats in the People's Republic of China, although the virus was not demonstrated to be an agent for RTI in humans [7]. These orthoreoviruses including Pulau virus, which was isolated from *Pteropus hypomelanus* urine samples from Tioman Island, an island locates off the eastern shore of Malaysia [8] and Xi River virus [7], were genetically and antigenetically related to Nelson Bay orthoreovirus, which was isolated from *Pteropus policephalus* heart blood samples from Nelson Bay, in the Hunter Region of New South Wales, Australia [9].

Here, we report an imported case of an RTI associated with an orthoreovirus, which is classified to the species of *Nelson Bay orthoreovirus*, in a patient who returned to Japan from Bali, Indonesia in November 2007. The isolate was named “Miyazaki-Bali/2007”.

## Materials and Methods

### Patient

The patient of the present study was a 38-year-old Japanese man, who visited Bali, Indonesia in November 2007. After returning to Japan, he presented to a local hospital with high fever, joint pain, sore throat, and cough.

### Virus isolation and identification

A throat swab specimen collected from the patient was mixed with viral transport medium, and transported to the Department of Microbiology, Miyazaki Prefectural Institute for Public Health and Environment. The throat swab sample was centrifuged for 10 min at 15000×g at 4°C, and the supernatant fractions were inoculated into the monolayers of the RD-18S, HEp-2, Vero, and CaCo-2 cell lines. Cells were cultured in Eagle's minimum essential medium supplemented with heat-inactivated 2% fetal bovine serum (MEM-2FBS). Cell morphologies were observed daily to detect the appearance of cytopathic effect (CPE). Virological examination was also performed to assess the presence of other respiratory viruses, including highly pathogenic avian influenza virus A H5N1 and severe acute respiratory syndrome coronavirus.

Furthermore, presence of viral RNA in the supernatant fractions of cell culture medium was evaluated by using the method, a rapid determination system of viral genome detection (RDV), as reported previously [10]. The RDV method, which was developed by our group, is a rapid method for the direct determination of viral RNA sequences without using the cDNA cloning step. The RDV method uses whole-genome amplification and direct nucleotide sequencing techniques, in which multiple steps; 1) effective destruction of cellular RNA and DNA for semipurification of viral particles, 2) effective elimination of DNA fragments by using a prefiltration column system and elution of small amounts of RNA, 3) effective synthesis of first- and second-strand cDNA library, 4) construction and amplification of a cDNA library, 5) construction of a second cDNA library, and 6) direct sequencing using optimized primers. The method is effective in the identification of unknown emerging viruses [10]–[15].

### Viruses

Orthoreovirus Miyazaki-Bali/2007 isolated in this study was used. Additionally, avian orthoreovirus [16], mammalian orthoreovirus 1 [17], and mammalian orthoreovirus 3 [18] were used for evaluation of migration pattern of Miyazaki-Bali/2007-dsRNA segments in comparison with those of these viruses.

### Electron microscopic analysis

The culture supernatant of Vero cells, in which CPE appeared, was used for electron microscopic observation. Samples were fixed with 4% glutaraldehyde, negatively stained with 2% phosphotungstic acid, and then observed using a JEM-1400 transmission electron microscope (JEOL Ltd, Tokyo, Japan).

### SDS-PAGE analysis of viral genome segment

Viral dsRNAs were purified as previously described [19]. RNA genomes were purified from each virus solution by using TRIzol (Life Technologies). Each of the purified RNA was mixed with LiCl at its concentration of 2 M, followed by incubation of the mixture at 4°C overnight. The mixture was then centrifuged at 14,000 rpm for 30 min for separation of ssRNA from dsRNA. The supernatant fraction containing dsRNA was treated with ethanol precipitation. Precipitated dsRNA was solubilized with water and separated by 10% SDS-polyacrylamide gel electrophoresis. Genome segments were visualized by ethidium bromide staining.

### Plaque assay for determination of infectious dose

Infectious dose of Miyazaki-Bali/2007 was determined by a plaque assay using Vero cell monolayers. Briefly, Vero cell monolayers were inoculated with each of serially diluted virus solutions and incubated for 1 hr for adsorption. The cell monolayers were washed with phosphate buffered saline solution and the cells were cultured with MEM-2FBS supplemented with 0.8% agarose for 3 days. Plaque was visualized by staining the cells with neutral red solution.

### Neutralization test

Virus solution containing approximately 100 plaque forming units was mixed with heat-inactivated and diluted serum samples for 1 h at 37°C. The mixtures were then inoculated onto a monolayer of Vero E6 cells. Cells were cultured for 4 days. The neutralization titer was defined as the reciprocal of the highest dilution at which no CPE was observed. When specific CPE appeared in the cells inoculated with serum samples, which were diluted 5 times with PBS, the serum was identified as neutralization activity negative.

### Nucleotide sequence determination

cDNA was synthesized by reverse transcription using random hexamer from dsRNA purified by using QIamp viral RNA kit (Qiagen, Germany). Partial genes of S1-, S2-, S3-, and S4-segments, which codes sigma C protein, major inner capsid protein, nonstructural replication (NS) protein, and major outer capsid protein, respectively, were amplified by RT-PCR. Primer sets were designed based on the nucleotide sequences of the conserved regions of the Melaka, HK23629/07, Kampar, and Sikamat orthoreoviruses. The nucleotide sequence of the genomes amplified was determined using the direct sequencing method.

### Phylogenetic analyses

The orthoreovirus nucleotide sequence data obtained in this study or from GenBank (Table 1) were phylogenetically analyzed using MEGA5.2 (PMID: 21546353). The nucleotide sequences of the open reading frames (ORFs) for sigma C protein (S1-segment), major inner capsid protein (S2-segment), sigma NS protein (S3-segment), or major outer capsid protein (S4-segment) extracted from GenBank (Table 1) were aligned using MUSCLE and program built into MEGA5.2. Evolutionary distances between amino acid sequences were estimated using the Poisson model and phylogenetic trees were constructed using the neighbor-joining method. The robustness of the trees was tested using 1000 bootstrap replication. The regions used for the phylogenetic analyses are shown in Table 1. The nucleotide sequences of the S1-, S2-, S3-, and S4-segments of Miyazaki-Bali/2007 are deposited in GenBank with the accession numbers of AB521793, AB521794, AB521795, and AB521796, respectively.

**Table 1:** Orthoreoviruses and accession numbers used in the present study.

Reovirus and strain	Accession No.			
	Sigma C protein (S1-segment, region used for phylogenetic analyses)	Major inner capsid protein (S2-segment, region used for phylogenetic analyses)	Sigma NS protein (S3-segment, region used for phylogenetic analyses)	Major outer capsid protein (S4-segment, region used for phylogenetic analyses)
Miyazaki-Bali/2007	AB521793 (550–1545)	AB521794* (1–1241)	AB521795 (4–1107)	AB521796* (9–1094)
Mammalian 1 strain Lang		L19774 (19–1275)		M13139 (33–1104)
Mammalian 2 strain D5/Jones	M10261 (14–1213)	L19775 (19–1275)		X60066 (33–1104)
Mammalian 3 strain Dearing	HM159619 (13–1380)	L19776 (19–1275)	HM159621 (28–1128)	K02739 (33–1112)
Mammalian 4 strain Ndelle	AF368035 (71–1387)	AF368036 (19–1275)		AF368037 (33–1104)
Avian strain 176	AF218358 (630–1610)	AF059716 (16–1266)	AF059724 (24–1127)	af059720 (31–1134)
Avian strain 138	AF218359 (630–1610)	AF059717 (16–1266)	AF059725 (24–1127)	AF059721 (31–1134)
Muscovy duck strain 89026	AJ310525 (1–810)	AJ278102 (16–1266)	AJ133122 (24–1127)	AJ006476 (31–1134)
HK23629/07	EU165526 (529–1501)	EU170365 (1–1178)	EU170366 (1–1046)	EU170367 (9–1078)
HK46886/09	JF803294 (529–1501)	JF803296 (1–1178)	JF803298 (1–1046)	JF803300 (9–1078)
HK50842/10	JF803295 (529–1501)	JF803297 (1–1178)	JF803299 (1–1046)	JF803301 (9–1078)
Kampar	EU448334 (527–1567)	EU448335 (16–1266)	EU448336 (29–1132)	EU44833 (30–1115)
Melaka	EF026043 (584–1570)	EF026044 (16–1266)	EF026045 (29–1132)	EF026046 (30–1115)
Nelson Bay	AF218360 (611–1582)	AF059718 (16–1266)	AF059726 (29–1132)	AF059722 (31–1116)
Pulau	AY357730 (582–1565)	AY357731 (16–1266)	AY357732 (29–1132)	AY357733 (31–1116)
Sikamat/MYS/2010	JF811580 (570–1568)	JF811581 (16–1266)	JF811582 (29–1132)	JF811583 (30–1115)
Xi river	GU188274 (611–1582)		GU188275 (29–1132)	
Baboon		AF059719 (17–1258)	AF059727 (23–1084)	AF059723 (31–1221)
Reptilian				AY238886 (32–1195)

*: When deposited, the virus was named “Miyazaki.” In the present study, it is named “Miyazaki-Bali/2007.”

### Surveillance of contacted persons

Serum samples were collected from 46 people who may have had contact with the patient (including family members, the patient's hospital caregivers, and workers in local health stations). Samples were subjected to neutralization antibody assay as described above. Information on symptoms after possible contact with the patient was collected.

### Ethical statement

Serum samples collected from the patient diagnosed as having orthoreovirus infection were obtained only for etiological analyses under the informed consent with the written document from him. Furthermore, travel history, clinical and laboratory data of the patient with the orthoreovirus infection described in this study were also disclosed under the written informed consent obtained through the easy-to-understand explanation including the human rights for him. Human rights of the patient were protected as much as possible.

Serum samples collected from the contact persons with the index patient were also collected only for detection of the neutralization antibody to the orthoreovirus isolated, Miyazaki-Bali/2007, under the written informed consent. All the protocols and procedures were approved by the Research and Ethical Committees for use of human subjects of the National Institute of Infectious Diseases, Tokyo, Japan (No. 452).

## Results

### Patient

A 38-year-old Japanese man visited Bali, Indonesia, with his wife on November 8, 2007. Eleven days later, on November 19, he showed symptoms of high fever, joint pain, sore throat, and cough. He returned to Miyazaki, Japan on November 21, and presented to a local hospital. The patient was hospitalized on the same day. At the time of presentation, the patient's vital signs were stable, he had no skin rashes or purpura and his throat was neither reddish nor swollen. A physical abdominal examination revealed no abnormal findings. A small lymph node in his right neck was palpable with mild tenderness. Chest X-ray examination showed no signs of lower respiratory tract infection.

Laboratory findings were as follows. Proteinuria and hematuria were negative. Peripheral total blood cell counts revealed that the patient's white blood cell, red blood cell, hemoglobin, and platelet counts were within normal ranges. Serum chemistry analyses showed normal values of total bilirubin, aspartate aminotransferase, alanine aminotransferase, lactate dehydrogenase, and amylase. No abnormalities were observed in serum electrolyte concentrations. Blood urea nitrogen and creatinine levels were normal. At 2.66 mg/dl, the patient's C-reactive protein was elevated in comparison to the normal range of 0.00–0.30 mg/dl.

On November 29, eight days after admission, all of the patient's symptoms resolved and he was discharged without any sequelae.

### Virus isolation and characterization

**Figure 1:**
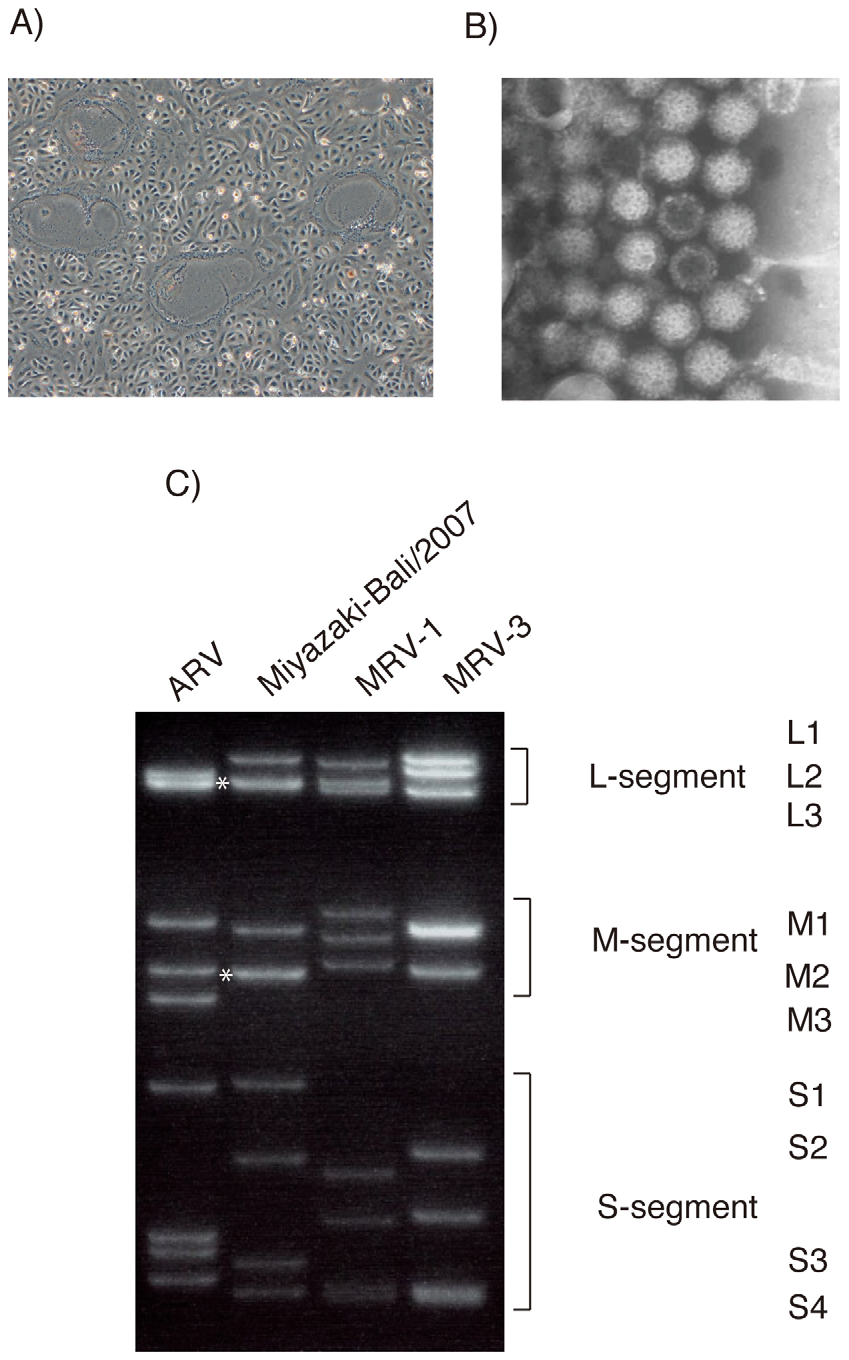
Characteristics of a novel orthoreovirus, Miyazaki-Bali/2007, CPE in Vero E6 cells induced by infection (A), electron microscopic morphology (B), and separation patterns of dsRNA separated by 10% SDS-PAGE electrophoresis (C) in comparison with those of ARV, MRV1 and MRV3. L2 and L3 segments, and M2 and M3 are considered to have co-migrated, forming one bond (*).

Cytopathic effect, characterized by syncytium formation, appeared in Vero E6 cells one day after inoculation with the throat swab specimens (Fig. 1A). Virological examination showed the patient to be seronegative for parainfluenza, mumps, and herpes viruses. Next, a rapid determination system of viral genome detection, which was developed in the authors' laboratory, was carried out with supernatants of CPE-positive cultures [10], revealing the presence of a virus genome sequence that was similar to that of the species of *Nelson Bay orthoreovirus* such as HK23629/07 and Melaka orthoreovirus [3], [6].

RT-PCR was performed against the isolated virus with primer sets designed specifically for the amplification of the S4 segments of HK23629/07. Genetic analysis indicated that the amplified DNA was 98% homologous to HK23629/07 (data not shown). Comparison of genome segments of the CPE agent by gel electrophoresis revealed an electropherotype that was almost identical to the previously reported electropherotypes of Melaka and Pulau orthoreoviruseses (Fig. 1C) [3].

Electron microscopic examination of the supernatant of the isolated virus revealed icosahedral particles resembling those of the family *Reoviridae*, genus *Orthoreovirus* (Fig. 1B) [3]. The particles possessed a mean diameter of approximately 75 nm.

Neutralization antibody titers rose significantly against the isolated virus; from 10 in the acute phase to 320 in the convalescent phase.

The CPE agent was identified as a species of the *Nelson Bay orthoreovirus* based on the above results and the virus was named “Miyazaki-Bali/2007.”

### Homology of nucleotide and amino acid sequences among the species of *Nelson Bay orthoreovirus*

Homology of nucleotide and amino acid sequences among the species of *Nelson Bay orthoreovirus* is summarized (Table 2). S1-segment of Miyazaki-Bali/2007 had the highest homology with being 97% to those of HK46886/09 and HK50842/10, which also seemed to have originated in Indonesia. Interestingly, S1-segment of Miyazaki-Bali/2007 showed 94% homology to that of Kampar virus originated in Malaysia, while it did 48–59% homology to those of the other orthoreoviruses in the genus of *Nelson Bay orthoreovirus*. S2-segment of Miyazaki-Bali/2007 had the highest homology with being approximately 92–94% in nucleotide sequences to those of HK23629/07, HK46886/09, and HK50842/10, which also seem to have originated in Indonesia [6], [20], while it showed 83–90% homology to those of Kampar, Melaka, Pulau, Sikamat, and Nelson Bay viruses. When the homology was evaluated using the nucleotide sequences of S3-segment, similar results were demonstrated (Table 2). The nucleotide sequence was conserved at the rate of more than 80% among the orthoreoviruses in the genus of *Nelson Bay orthoreovirus*. The homology of Miyazaki-Bali/2007 S4-segment was approximately 94% to those of HK23629/03, HK46886/09, and HK50842/10 and less than those to other orthoreoviruses. The homology pattern was almost the same as that observed in S2-segment.

**Table 2:** Homology in nucleotide and amino acid sequences of Sigma C protein (S1-segment), major inner capsid protein (S2-segment), sigma NS protein (S3-segment), and major outer capsid protein (S4-segment) between the orthoviruses of *Nelson Bay* group as well as avian, baboon and mammalian orthoreoviruses.

Sigma C (S1-segment) [Nucleotide identity (right column) and amino acid (column below)]	Miyazaki-Bali/2007	HK23629/07	HK46886/09	HK50842/10	Kampar	Melaka	Nelson_Bay	Pulau	Sikamat/MYS/2010	Xi river	Avian_strain_138	Mammalian_2_strain_D5/Jones
Miyazaki-Bali/2007		56.6	97.5	97.1	93.9	56.8	48.0	58.5	57.3	48.5	36.2	23.7
HK23629/07	54.9		58.0	57.8	56.3	63.9	45.4	63.1	64.9	47.4	35.4	21.7
HK46886/09	97.8	56.1		99.5	91.7	54.7	46.0	56.2	55.1	46.5	35.1	23.3
HK50842/10	97.2	55.5	99.3		91.5	54.7	46.1	56.2	55.1	46.6	35.1	23.3
Kampar	93.6	55.2	91.5	90.9		56.1	48.2	58.6	56.5	48.1	36.6	24.0
Melaka	56.7	64.3	54.6	54.9	55.5		48.1	75.2	94.9	50.3	36.3	21.9
Nelson_Bay	42.2	38.9	40.7	41.0	41.6	40.5		48.2	48.2	60.0	39.3	23.5
Pulau	52.2	62.5	50.1	50.1	51.3	78.9	38.4		75.0	48.6	37.9	23.6
Sikamat/MYS/2010	56.9	65.6	54.8	55.1	55.4	95.1	40.0	78.9		49.9	36.2	21.7
Xi river	41.9	39.5	40.4	40.7	41.0	44.2	57.8	38.7	43.3		38.1	24.1
Avian_strain_138	23.6	23.0	22.7	22.7	23.0	23.5	23.9	25.3	24.4	23.9		23.2
Mammalian_2_strain_D5/Jones	12.6	11.7	12.2	12.0	12.8	11.7	10.0	11.2	11.6	10.2	10.4	

Major inner (S2-segment)	Miyazaki-Bali/2007	HK23629/07	HK46886/09	HK50842/10	Kampar	Melaka	Nelson_Bay	Pulau	Sikamat/MYS/2010	Avian_strain_138	Baboon	Mammalian_ 1_strain Lang
Miyazaki-Bali/2007	-	94.2	94.2	91.8	87.1	87.2	83.1	89.8	86.8	56.1	41.3	41.6
HK23629/07	94.1	-	99.4	96.9	82.9	82.9	78.7	85.4	82.6	53.0	39.4	39.8
HK46886/09	94.1	99.4	-	96.8	82.8	82.6	78.8	85.3	82.6	53.0	39.5	39.8
HK50842/10	94.1	99.4	99.4	-	82.3	82.2	79.2	84.8	82.1	53.3	39.7	40.1
Kampar	97.5	92.5	92.5	92.5	-	95.5	86.0	92.1	99.5	59.4	42.4	43.6
Melaka	98.0	93.0	93.0	93.0	99.0	-	85.8	92.2	95.2	59.1	42.1	43.0
Nelson_Bay	97.1	92.0	92.0	92.0	97.8	98.0	-	86.9	86.0	59.4	43.2	43.8
Pulau	97.5	92.5	92.5	92.5	98.0	98.5	97.1	-	91.9	58.5	42.4	43.2
Sikamat/MYS/2010	97.3	92.5	92.5	92.5	99.2	98.7	97.5	97.8	-	59.3	42.4	43.3
Avian_strain_138	58.6	55.2	55.5	55.7	59.8	59.6	60.0	59.6	59.6	-	40.5	42.2
Baboon	29.4	28.7	28.7	28.7	30.6	30.6	31.1	30.6	30.3	28.9	-	40.7

Sigma NS (S3-segment)	Miyazaki-Bali/2007	HK23629/07	HK46886/09	HK50842/10	Kampar	Melaka	Nelson_Bay	Pulau	Sikamat/MYS/2010	Xi river	Avian_strain_138	Baboon
Miyazaki-Bali/2007		94.2	85.1	85.0	89.6	89.7	86.5	90.1	90.0	90.0	52.2	38.8
HK23629/07	94.5		89.8	89.7	84.4	84.2	81.7	84.8	84.6	84.6	49.0	36.1
HK46886/09	93.4	98.8		99.9	89.3	89.0	81.7	89.8	89.0	88.7	49.3	35.9
HK50842/10	93.4	98.8	100		89.2	88.9	81.6	89.7	88.9	88.6	49.4	35.8
Kampar	98.3	92.9	93.4	93.4		93.9	86.8	98.6	96.1	93.0	52.7	38.2
Melaka	98.9	93.4	94.0	94.0	98.9		85.9	94.1	93.6	96.0	52.2	38.8
Nelson_Bay	96.7	91.2	92.0	92.0	96.7	97.2		87.3	86.7	86.3	52.6	39.4
Pulau	98.6	93.1	93.7	93.7	99.1	99.1	97.0		96.7	93.5	52.7	38.3
Sikamat/MYS/2010	98.9	93.4	94.0	94.0	98.9	99.4	97.2	99.1		92.9	52.0	38.1
Xi river	99.1	93.7	93.7	93.7	98.6	99.7	97.0	98.9	99.1		52.5	38.6
Avian_strain_138	50.1	47.1	47.1	47.1	49.8	49.8	50.1	50.1	50.1	49.8		37.7
Baboon	25.8	23.3	23.3	23.3	25.8	26.0	26.0	25.8	25.8	26.0	26.1	

Outer capsid (S4-segment)	Miyazaki-Bali/2007	HK23629/07	HK46886/09	HK50842/10	Kampar	Melaka	Nelson_Bay	Pulau	Sikamat/MYS/2010	Avian_strain_138	Baboon	Mammalian_ 1_strain Lang
Miyazaki-Bali/2007	-	95.5	93.6	93.7	81.7	81.6	76.7	78.9	81.2	36.9	33.3	30.6
HK23629/07	98.3	-	97.4	97.7	82.6	82.6	77.7	80.3	82.2	37.3	32.3	30.4
HK46886/09	98.6	99.1	-	97.9	82.7	82.7	77.4	80.5	81.8	36.9	32.2	30.6
HK50842/10	98.3	98.8	99.1	-	82.6	82.3	77.6	80.1	81.7	37.2	32.1	30.6
Kampar	96.6	95.8	96.1	96.3	-	95.7	83.2	91.0	92.8	38.9	34.0	33.3
Melaka	98.0	96.9	97.2	97.5	97.5	-	83.8	94.1	96.1	40.0	34.6	34.1
Nelson_Bay	93.3	92.2	92.5	92.5	91.9	93.3	-	82.3	83.5	38.8	33.6	32.4
Pulau	92.7	91.6	91.9	92.2	91.6	93.6	88.6	-	92.9	38.9	34.3	33.5
Sikamat/MYS/2010	98.3	97.2	97.5	97.7	96.6	98.6	93.6	93.6	-	39.9	34.8	33.9
Avian_strain_138	34.8	34.0	34.0	33.7	34.8	34.8	35.1	34.5	34.5	-	30.0	36.5
Baboon	17.9	17.1	17.1	17.1	17.9	18.4	17.6	17.6	18.4	17.8	-	32.4
Mammalian_1_strain_Lang	17.0	16.2	16.2	16.2	16.7	16.7	17.0	15.6	17.0	15.8	11.2	-

### Phylogenetic analyses

**Figure 2:**
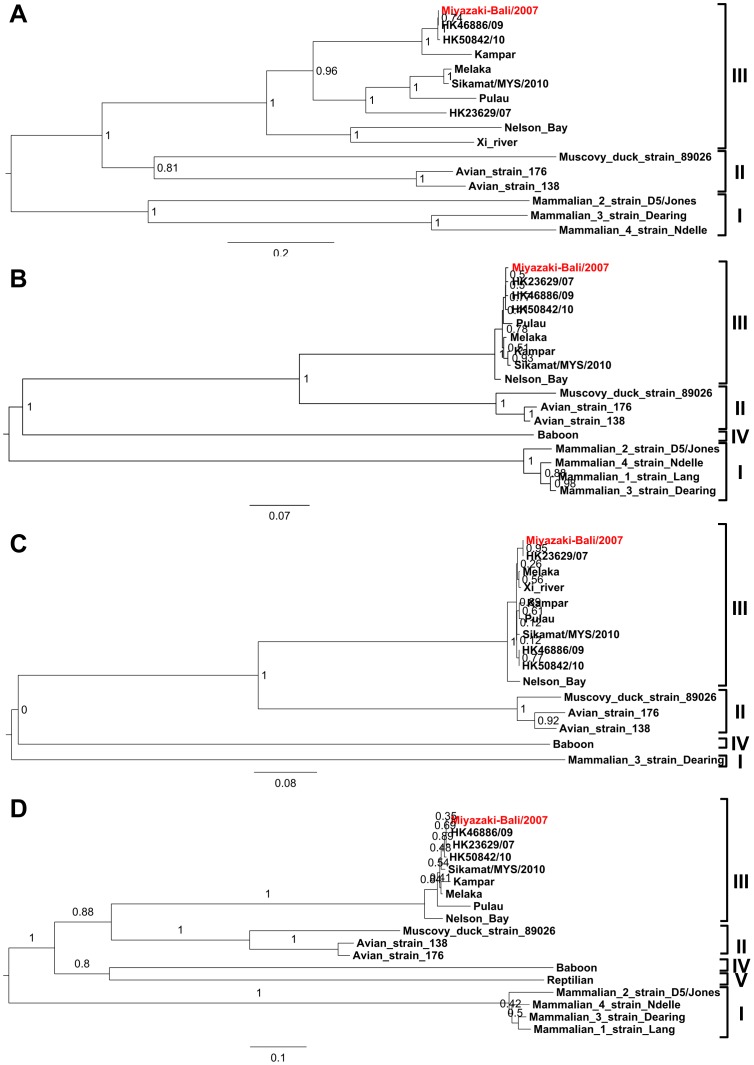
Phylogenetic trees showing the phylogenetic positions of the Miyazaki-Bali/2007 strain and other orthoreovirus strains based on deduced amino acid sequence of the sigma C protein (A), major inner capsid protein (B), sigma NS protein (C), and major outer capsid protein (D) listed in Table 1. Bootstrap values for each node are shown by the robustness of the tree. Miyazaki-Bali/2007, the strain isolated in the patient of the present study, is indicated in red font.

Miyazaki-Bali/2007, together with the other species of *Nelson Bay orthoreovirus*, formed a cluster that was independent to avian, mammalian, and baboon reoviruses (Fig. 2). Miyazaki-Bali/2007 was found to be most closely related to HK23629/07, HK46886/09, and HK50842/10 based on the phylogenetic analyses with S2- and S4-segments. [6], [20]. However, Miyazaki-Bali/2007 did not form a subcluster with HK23629/07, when analyzed with S1-segment sequences (Fig. 2A). Furthermore, Miyazaki-Bali/2007 formed a subcluster with HK23629/07, but not with HK46886/09 and HK50842/10, when analyzed with S3-segment sequences (Fig. 2C), although all the 4 viruses including Miyazaki-BaliHK23629/07, HK46886/09, and HK50842/10 originated from Indonesia.

### Surveillance on contacted persons

Serum samples collected from all the persons, who came into contact with the index patient, showed negative activity in the neutralization antibody test, in spite of the fact that one of the patient's family members and three of his caregivers in the hospital had experienced fever and sore throat within one week of the contact. These data suggest that human-to-human transmission of the virus did not easily occur.

## Discussion

The first orthoreovirus of bat origin, Nelson Bay orthoreovirus, was isolated in 1968 from the heart blood of a grey-headed flying fox (*Pteropus poliocephalus*), a species of fruit bat, in New South Wales, Australia, followed by the isolation of Pulau virus, which was also isolated from a fruit bat, specifically a small flying fox (*Pteropus hypomelanus*). Pulau virus was found to be serologically and genetically related to Nelson Bay orthoreovirus [8], [9], [21]. At the time of isolation, it was not yet known whether these bat orthoreoviruses were capable of infecting or causing diseases in humans and/or other animals.

An orthoreovirus, Melaka virus, was first reported as a causative agent for RTI in Malaysia [3]. This was followed by several reports describing RTI in humans being caused by the viruses with similar characteristics [4]–[6], [20]. The characteristics of these sporadic outbreaks are summarized in Table 3.

**Table 3:** Summary of the orthoreoviruses belong to the species of *Nelson Bay orthoreovirus* isolated from humans with RTI.

Name of isolate	Isolation year and month	Location of isolation (origin)	Human-to-human transmission episode	Patient characteristics/major symptoms	Reference
Miyazaki/Bali	2007.11	Miyazaki, Japan (Bali, Indonesia)	Not demonstrated (including in close contact)	38-year-old man/fever, cough, sore throat	This study
Melaka virus	2006.03	Melaka/Malaysia	Not demonstrated	39-year-old man/fever, cough, sore throat, headache, myalgia, malaise, loss of appetite, general body weakness, sore throat with swallowing difficulty	[3]
Kampar virus	2006.08	Kampar/Malaysia	Possibly demonstrated to medical doctor (nosocomial infection) and to his wife	54-year-old man/fever, chills, rigor, cough, sore throat, headache, myalgia, body ache, severe malaise, vomiting, diarrhea, abdominal pain, loss of appetite	[4]
HK23629/07	2007.04	Hong Kong/Bali, Indonesia	Not described	51-year-old man/fever, sore throat, myalgia, watery diarrhea	[6], [20]
Sikamat	2010.03	Sikamat/Malaysia	Possibly to patient's wife and one of his sons	46-year-old man/sore throat, myalgia, headache, photophobia	[5]
HK46886/09	2009.07	Hong Kong/Bali, Indonesia	Not described	26-year old woman/influenza like illness	[20]
HK50842/10	2010.06	Not described/Indonesia	Not described	29-year-old woman/influenza like illness	[20]

This is the sixth report describing a patient infected with an orthoreovirus classified to the species of *Nelson Bay orthoreovirus*. Three patients were infected in Malaysia, and the other 4 patients including the patient in the present study were infected in Indonesia and imported to Hong Kong [6], [20] or Miyazaki. It is noteworthy that both the present and 2 of the Hong Kong cases were infected with their respective viruses in Bali. The phylogenetic analyses indicated a close association among these isolates, when analyzed with S2- and S4-segments (Fig. 2B and 2D). The association pattern based on the S2- and S4-segments' sequences was different from those based on the S1- and S3-segments' sequences (Fig. 2). The cause behind this difference might be the reassortment event among these orthoreoviruses in the endemic regions. Further study is needed to confirm this assumption. Although the travel information on the patient infected with HK23629/07 was not described in detail [6], it was described that the patient was infected in April, 2007 [20], while the patient in the present study was infected in November, 2007. There seemed to be no direct epidemiological link between the two patients. An epidemiological investigation to clarify the epidemiology of the infections associated with the species of *Nelson Bay orthoreovirus* among residents in Southeastern Asia would be valuable.

We found no evidence of human-to-human transmission of the virus among the family members and hospital caregivers, who were in contact with the patient. Given that only a small number of possible human-to-human transmission episodes were reported in the sporadic outbreaks of Melaka, Kampar, and Sikamat viruses [3]–[5], currently the risk of human-to-human infection of these orthoreoviruses would seem to be low.

The antigenicity of Miyazaki-Bali/2007 virus was not compared with other orthoreoviruses in the genus of *Nelson Bay orthoreoviruses*. While neutralization activity was examined during the convalescent phase in the Miyazaki-Bali/2007 case of the present study, further study is needed to clarify the neutralization activity of the serum sample collected from the patient in the convalescent phase against other species of *Nelson Bay orthoreoviruses*.

The orthoreoviruses isolated from patients with RTI were classified to the species of *Nelson Bay orthoreovirus* are more closely associated in the phylogenetic analyses with Pulau virus than Nelson Bay virus (Fig. 2). Thus far, there is no direct evidence of bat origin in the reported cases. However, epidemiological studies that were performed in each of the reports, with the exception of the Hong Kong case, indicated the potential for bat exposure before the onset of the disease [3]–[5]. That each of the Melaka, Kampar, and Sikamar virus patients resided in areas, in which fruit bats were present, supports this possibility. In the Hong Kong case, where the patient was also infected in Bali, no detailed information was recorded on the environment, in which the patient resided [6]. One patient, who might be infected in Bali, Indonesia, in 2009, visited a safari park and entered bat caves during the stay in Bali [20]. This suggests the fruit bat as a possible vector in these orthoreovirus infections. However, the infection mode of the species of *Nelson Bay orthoreoviruses* in humans is still unknown and should be a subject for prospective studies.

Miyazaki-Bali/2007 orthoreovirus caused respiratory tract infection in mouse model, in which mice were infected with the virus through intranasal inoculation route (data not shown, but presented at the 56th Annual Meeting of The Japanese Society for Virology, Okayama, Japan, in October, 2008 with the presentation number “3C18”). Furthermore, the virus was isolated from and detected at the lesions in the lung. It suggests that Miyazaki-Bali/2007 orthoreovirus caused RTI in the patient.

Bats have recently been identified to be the reservoir hosts of a variety of viruses responsible for severe zoonotic virus infectious disease outbreaks with very high mortality [22]. Examples of such infections are encephalitis caused by Hendra virus [23], [24], Nipah virus [25] (family *Paramyxoviridae*), Menangle virus (another new paramyxovirus) [26], [27], and Australian bat lyssavirus (family *Rabdoviridae*) [22], [28]. Marburgvirus (family *Filoviridae*), the etiological agent for viral hemorrhagic fever in the continent of Africa, has been isolated from Egyptian fruit bats (*Rousettus aegyptiacus*), suggesting that fruit bats are a viral reservoir for marburgvirus [29]. The virus genome of ebolavirus, another genus of family *Filoviridae* and also an etiological agent for hemorrhagic fever, was detected in serum samples collected from three species of fruit bats: *Hypsignathus monstrosus*, *Epomops franqueti*, and *Myonycteris torquata* [30]. This suggests that these species might serve as a reservoir for ebolavirus.

In summary, the authors described an imported case of an RTI associated with an orthoreovirus, which was classified to the species of *Nelson Bay orthoreovirus*, from Bali, Indonesia, to Miyazaki, Japan, that occurred in 2007. The virus was closely related to the viruses isolated from patients possibly infected with the orthoreoviruses in Indonesia. Prospective surveillance of the *Nelson Bay* orthoreovirus species group should be performed in Southeast Asia.
